# The relative influence of habitat amount and configuration on genetic structure across multiple spatial scales

**DOI:** 10.1002/ece3.1325

**Published:** 2014-12-05

**Authors:** Katie L Millette, Nusha Keyghobadi

**Affiliations:** Department of Biology, Western UniversityLondon, Ontario, Canada, N6A 5B7

**Keywords:** Dispersal, gene flow, habitat structure, isolation, landscape genetics

## Abstract

Despite strong interest in understanding how habitat spatial structure shapes the genetics of populations, the relative importance of habitat amount and configuration for patterns of genetic differentiation remains largely unexplored in empirical systems. In this study, we evaluate the relative influence of, and interactions among, the amount of habitat and aspects of its spatial configuration on genetic differentiation in the pitcher plant midge, *Metriocnemus knabi*. Larvae of this species are found exclusively within the water-filled leaves of pitcher plants (*Sarracenia purpurea*) in a system that is naturally patchy at multiple spatial scales (i.e., leaf, plant, cluster, peatland). Using generalized linear mixed models and multimodel inference, we estimated effects of the amount of habitat, patch size, interpatch distance, and patch isolation, measured at different spatial scales, on genetic differentiation (*F*_ST_) among larval samples from leaves within plants, plants within clusters, and clusters within peatlands. Among leaves and plants, genetic differentiation appears to be driven by female oviposition behaviors and is influenced by habitat isolation at a broad (peatland) scale. Among clusters, gene flow is spatially restricted and aspects of both the amount of habitat and configuration at the focal scale are important, as is their interaction. Our results suggest that both habitat amount and configuration can be important determinants of genetic structure and that their relative influence is scale dependent.

## Introduction

The abundance and distribution of habitat in a landscape (i.e., habitat structure) is one of the most influential factors driving species abundance patterns over space and time (Turner et al. 2001). There are two distinct and quantifiable components of habitat structure: habitat amount and configuration. Habitat amount quantifies suitable habitat in a landscape, while habitat configuration describes the spatial characteristics and arrangement of habitat patches. Both habitat amount and configuration can influence ecological (e.g., behavior, dispersal, reproduction) and evolutionary processes (e.g., genetic drift, gene flow), which in turn contribute to the long-term sustainability of natural populations and biodiversity (MacArthur and Wilson 1967; Diamond 1975).

The relative importance of the amount of habitat versus its configuration for ecological processes is a long-standing issue in landscape ecology (Turner 2005), particularly for understanding the effects of habitat fragmentation on species and ecosystems (Fahrig 2003). With fragmentation, the physical breaking up of habitat patches (fragmentation per se) typically occurs simultaneously with habitat loss, making it difficult to assess the extent to which species abundance and diversity are responding to changes in the spatial configuration of habitat patches versus simply the amount of habitat (Fahrig 2003).

Some ecological field studies have been able to measure habitat amount and configuration independently (e.g., Villard et al. 1999; Schmiegelow and Monkkonen 2002; Cushman and McGarigal 2004) or have manipulated them experimentally (Bonin et al. 2011; With and Pavuk 2011). Overall, the results of these studies indicate that spatial configuration of habitat often contributes little to species occupancy, abundance, and distribution patterns, particularly when the amount of habitat in the landscape is high. However, as habitat becomes less abundant (e.g., 10–30%; Radford et al. 2005), the configuration of the habitat becomes increasingly important (McGarigal and McComb 1995; Trzcinski et al. 1999). Thus, there can be a strong, but highly context-dependent influence of habitat configuration on the distribution and abundance of species, a conclusion further supported by theoretical and simulation studies (With and Crist 1995; Hill and Caswell 1999; Fahrig 2002; Flather and Bevers 2002).

The relative influence of, and potential interactions among, aspects of habitat amount and configuration on population genetics remain largely unexplored in empirical systems. To date, relevant investigations are limited to simulation studies which, in contrast to the aforementioned ecological studies, suggest that habitat configuration is more important than habitat area in determining genetic differentiation among populations. In simulation modeling of red-cockaded woodpecker, habitat fragmentation per se strongly affected effective population size and *F*_ST_ values (Bruggeman et al. 2010). Cushman et al. (2012) similarly conclude that habitat configuration variables, particularly habitat patch cohesion, correlation length, and aggregation index, are stronger determinants of genetic differentiation than habitat area. These results are not surprising given that gene flow and genetic drift are key processes determining levels of neutral genetic differentiation, and gene flow is expected to be a function of isolation, while drift in many cases is a function of local patch size (Wright 1943, 1951; Frankham 1997). Indeed, a strong theoretical basis for expecting habitat configuration to be an important determinant of genetic differentiation has led to much empirical research that focuses specifically on quantifying effects of patch size and isolation on genetic diversity and differentiation (Frankham 1997; Holmes et al. 2013). In such studies, the amount of habitat in the broader landscape is not generally considered, although it may influence genetic structure through stepping stone effects and by determining regional effective population size.

The relative influence of habitat amount and configuration on genetic structure likely varies with spatial scale as the processes determining genetic structure (e.g., reproductive behavior, dispersal, genetic drift) may operate at unique scales (Wiens 1989; Balkenhol et al. 2009; Anderson et al. 2010). Currently, there are no empirical evaluations of the relative influence of aspects of habitat amount and configuration on genetic structure in natural systems; nor do we have a strong understanding of how the effects of these factors change across spatial scales. Here, we begin to address these knowledge gaps, taking advantage of unique properties of the pitcher plant midge, *Metriocnemus knabi* Coquillett 1904 (Diptera, Chironomidae), and its habitat as a study system.

*Metriocnemus knabi* larvae are found exclusively within fluid-filled leaves of the purple pitcher plant, *Sarracenia purpurea* L., throughout patchy peatland habitats across eastern North America. The pitcher plant phytotelma represents an ecological microcosm used to address questions of population regulation, community interactions, and ecosystem processes (Addicott 1974; Srivastava et al. 2004; Kadowaki et al. 2012). Multiple leaves are found in each pitcher plant, and the plants tend to grow in clusters, likely as a result of subterranean rhizome growth and short seed dispersal (∼5 cm, Ellison and Parker 2002). Thus, the habitat of *M. knabi* is clearly defined by *S. purpurea* as discrete habitat patches that are hierarchically nested at several spatial scales (leaf, plant, cluster, and peatland). The abundance and distribution of leaves within pitcher plants, plants within clusters, and clusters within peatlands vary widely, such that various combinations of habitat amount and configuration occur naturally at each scale.

At temperate latitudes, *M. knabi* is univoltine and adults emerge in late spring. Little is known about the adult stage, although adults are small (∼3 mm in length) and likely have weak flight abilities (Knab 1905; Wiens 1972; Krawchuk and Taylor 2003; pers. obs. K. L. Millette). Females deposit eggs within pitcher leaves and multiple larvae (up to ∼15 individuals) can be found developing within a single leaf in late summer (Giberson and Hardwick 1999). Larvae of a flesh fly, *Fletcherimyia fletcheri*, and mosquito, *Wyeomyia smithii*, also develop exclusively within *S. purpurea*. All three insects have a commensal relationship with the plant, which provides a suitable aquatic environment and food from trapped, decomposing prey (Heard 1994). Although the plant is not dependent on the larvae, their presence contributes to enhanced nutrient availability (Bradshaw and Creelman 1984).

For all three pitcher plant insects, habitat structure influences larval abundance at several spatial scales (Krawchuk and Taylor 2003). In general, habitat configuration has a significant effect on larval abundance, regardless of the amount of habitat in the surrounding landscape; patch size is the most important configuration metric at distances within the dispersal range of individuals (i.e., leaf, plant), while patch isolation becomes important at broader scales (i.e., cluster and peatland; Krawchuk and Taylor 2003). Previous population genetic analyses on *M. knabi* indicate significant genetic structuring at all spatial scales (i.e., among individuals collected in different leaves, plants, clusters, and peatlands) with greater differentiation at the higher spatial scales (i.e., cluster, peatland; Rasic and Keyghobadi 2012). In addition, broad-scale landscape variables such as pitcher plant density and peatland size account for approximately 50% of the genetic differentiation among individuals from different leaves (Rasic and Keyghobadi 2012). Therefore, background evidence indicates that *M. knabi* larval abundance responds differentially to habitat amount and configuration depending on spatial scale and that cross-scale effects of habitat structure on genetic differentiation also occur. While Rasic and Keyghobadi (2012) examined generally the genetic structure of *M. knabii* across spatial scales, including effects of some landscape variables, they did not measure or evaluate independent metrics of habitat amount and configuration at any scale.

In this study, we evaluate the relative effects of patch size, interpatch distance, and the amount of habitat in the local landscape surrounding sampling locations on genetic differentiation in *M. knabi* across three spatial scales: among samples from different leaves within single pitcher plants (plant scale), from different plants within clusters (cluster scale), and from different clusters within peatlands (peatland scale). Each plant, cluster, and peatland can be considered a replicate “landscape” from which we have sampled multiple habitat patches. At each scale, we estimate genetic differentiation among the sampled patches within each “landscape”, and relate measures of differentiation to select habitat amount and configuration metrics. Specifically, we focus on the size and distance among sampled habitat patches, which are key configuration metrics most commonly measured in genetic studies of habitat fragmentation (Frankham 1997; Holmes et al. 2013). Unlike most previous studies, we also consider the effect of the total amount of habitat in the local landscape. Furthermore, the nested spatial scales of habitat allow us to investigate whether habitat amount or isolation at broader spatial scales than the focal scale contribute to patterns of genetic differentiation. Thus, we ask the following questions: (1) Does genetic differentiation depend only on the size and distances among sampled patches, or does the amount of habitat in the landscape matter? (2) Does the relative influence of these aspects of habitat configuration and habitat amount change with spatial scale? and (3) Is there evidence of cross-scale effects of habitat amount or configuration on genetic differentiation?

## Materials and Methods

### Study area and peatlands

**Figure 1:**
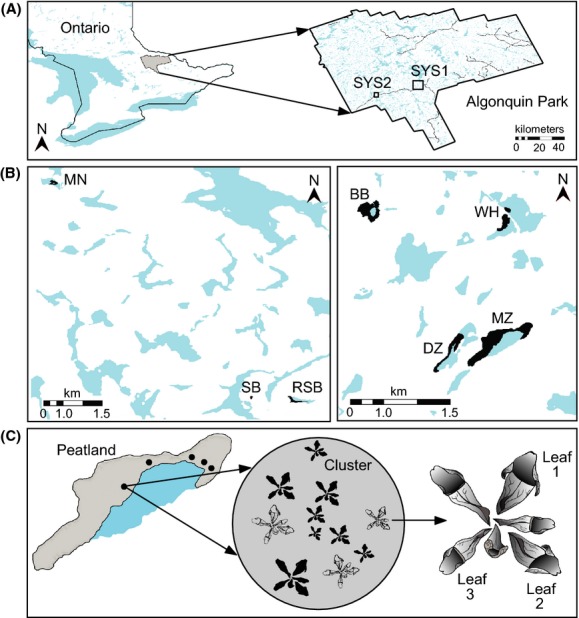
Sampling map of *Metriocnemus knabi*. (A) Larvae were sampled from two systems of peatlands (SYS1, SYS2) in Algonquin Provincial Park (Ontario, Canada). (B) System 1 consists of Minor Lake (MN), Roadside (RSB), and Spruce (SB) peatlands. System 2 consists of Buggy (BB), Dizzy Lake (DZ), Mizzy Lake (MZ), and Wolf Howl (WH) peatlands. (C) Within each peatland, 3–5 clusters of plants were arbitrarily selected. Within each cluster, three plants were chosen and larvae were removed from three leaves per plant.

The study was conducted in Algonquin Provincial Park, Ontario, Canada (Fig.1), in a transition zone between southern deciduous and northern coniferous forest. The predominant land cover is forest, within which peatlands (fen or bog-like environment; Gore 1983) are patchily distributed. As a result of poor drainage and accumulated plant material, peatlands are characteristically low in pH and oxygen and harbor a distinctive plant community that includes the carnivorous *S. purpurea*.

### Sampling

*Metriocnemus knabi* larvae were sampled in August 2011 at four nested spatial scales: leaf, plant, cluster, and peatland and replicated in two areas or “systems” approximately 25 km apart (Fig.1). Within each system, 3–4 peatlands were selected and 3–5 clusters of plants were sampled per peatland. A cluster was defined as a 5-m-radius area containing ≥10 pitcher plants, centered on the point of highest pitcher plant density. Three plants were haphazardly selected within a cluster, and larvae were removed from three leaves per plant (Fig.1). The locations of each cluster's center and each sampled plant were recorded using a high-accuracy (<30 cm) GPS receiver (Trimble GeoXH, Sunnyvale, CA; Table S1).

### Microsatellite genotyping

Larvae were removed from *S. purpurea* leaves, sorted, and preserved in 95% ethanol. Genomic DNA was extracted from single larvae using the DNeasy tissue extraction kit (Qiagen, Germantown, MD). Individuals were genotyped at 11 microsatellite loci (Rasic et al. 2009) and the 10 *μ*L multiplexed polymerase chain reactions (PCR), thermal cycling, and fragment analysis protocols followed that of Rasic and Keyghobadi (2012).

### Preliminary genetic data analyses

Loci were assessed for neutrality using LOSITAN software (Antao et al. 2008), which tests for loci potentially under selection using an *F*_ST_-based detection method (Beaumont and Nichols 1996). An island-model coalescent simulation of mutation-drift equilibrium was performed to generate the sampling distribution of single-locus *F*_ST_ values. The presence of significant outliers was tested using 50,000 permutations while assuming peatland population substructure (i.e., seven subpopulations) and an infinite allele mutation model. The mean number of alleles (*N*_A_), observed (*H*_O_), and expected (*H*_E_) heterozygosities were calculated across loci and samples for each plant, cluster, and peatland using GenAlEx version 6.4.1 (Peakall and Smouse 2006).

Full siblings represent individuals that have developed from eggs of a single clutch and the spatial distribution of full-sibling larvae, which do not disperse among leaves, therefore reflects the oviposition behavior of adult females. Relationships between pairs of larvae were assessed using ML-Relate (Kalinowski et al. 2006). Full-sibling (FS), half-sibling (HS), parent-offspring (PO), and unrelated (U) relationships were tested for all pairs of individuals sampled from within the same leaf, in different leaves of the same plant, in different plants of the same cluster, and in different clusters of the same peatland using a 95% confidence set and 1000 randomizations. As PO relationships are not possible for larvae collected within a single season, putative PO relationships were treated as FS. If an alternative relationship with a high likelihood was identified by the confidence set for each FS and/or PO relationship, the FS/PO relationship was tested against the alternative using a likelihood ratio test and 1000 simulated random genotype pairs (Kalinowski et al. 2006). Pairwise comparisons between individuals from the same lower level (e.g., plant) were removed when assessing relationships at higher levels (e.g., cluster).

A hierarchical analysis of molecular variance (AMOVA) was conducted to assess the distribution of genetic variation across all spatial scales in both systems. Variance components and hierarchical *F*-statistics were computed in R (version 2.14.1, R Core Team 2013) using the hierfstat package (Goudet 2005). Significance of variance components and *F*-statistics among leaves, plants, clusters, and peatlands in each system was tested using 1000 permutations and *α *= 0.05. In System 1, genetic variation was assessed across the three peatlands, 11 clusters, 33 plants, and 99 leaves (447 individuals). The large sample size of System 2 (four peatlands, 19 clusters, 57 plants, 169 leaves, 752 individuals) exceeded the computational limit of the hierfstat package, so variance components were assessed by removing one peatland at a time and averaging the resulting values.

### Genetic differentiation among sampled patches: response variable at each scale

Genetic differentiation was measured at the plant, cluster, and peatland scales using Weir and Cockerham (1984) estimates of *F*_ST_ in GenAlEx (Peakall and Smouse 2006). At the plant scale, *F*_ST_ was estimated among the three sampled leaves for each plant separately. Only plants containing a minimum of three individuals per leaf were included (*n *=* *65 plants). Similarly, at the cluster and peatland scales, *F*_ST_ was estimated among the three sampled plants within each cluster, and among the 3–5 sampled clusters within each peatland, respectively. At the cluster scale, only plants containing 9–15 individuals per plant (i.e., 3–5 individuals per leaf) were included (*n *=* *29 clusters). At the peatland scale, each cluster contained 27–45 individuals (*n *=* *7 peatlands). These *F*_ST_ values were subsequently used as the response variables in models describing effects of habitat amount and configuration on genetic differentiation. Significance of *F*_ST_ was assessed using 9999 permutations and *α *= 0.05. Note that this response variable (*F*_ST_) represents a node-based estimate of genetic differentiation. Thus, at the plant scale, a single value of *F*_ST_ was estimated among the three sampled leaves within each plant and the total sample size is the number of plants. This approach is different from most landscape genetic studies where the response variable is typically a pairwise measure of genetic differentiation. Unlike pairwise values, node-based estimates are not inherently interdependent (Legendre and Fortin 2010).

### Habitat configuration: patch size, interpatch distance, and patch isolation

**Figure 2:**
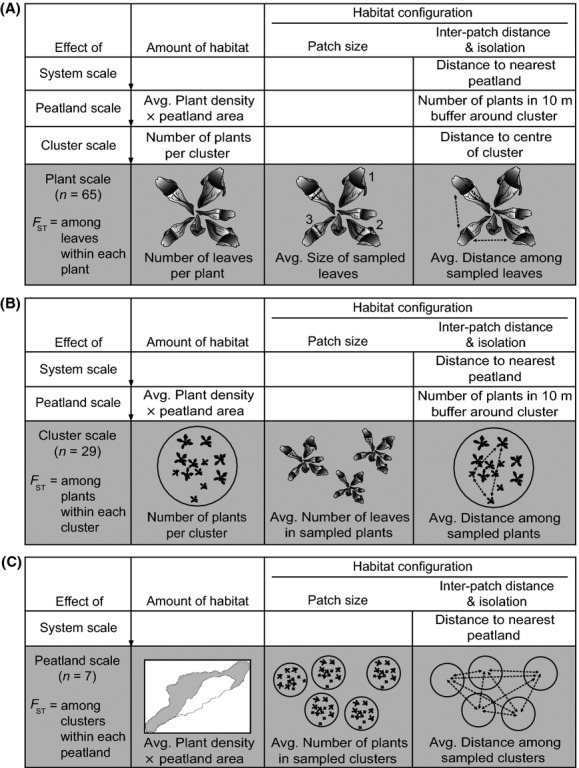
Summary of habitat amount and configuration measurements included in models at the (A) plant, (B) cluster, and (C) peatland scales.

The patch size of leaves (*S*_lf_) was measured as the length of the widest part of the pitcher vessel as this metric was a strong predictor of the leaf's potential volume (*R*^2^ = 0.92, *P *<* *0.001; Fig. S1; Table S2). Patch size of plants (*S*_pl_) and clusters (*S*_cl_) were measured as the number of leaves per plant, and the number of pitcher plants per cluster, respectively (Table S3). At each scale, the sizes of the three sampled patches (leaf, plant, or cluster) within each study unit (i.e., each “landscape”) were averaged to give a single patch size metric (*S*_lf_, *S*_pl_, *S*_cl_; Fig.2; Table S3).

At the plant scale, interpatch distance was measured in the field as the average distance among the three sampled leaves in each plant (*D*_lf_; Fig.2; Table S3), while at the cluster scale, interpatch distance was the average distance among the sampled plants (*D*_pl_; measured in the field). As clusters of plants were centered on the point of highest plant density and tend to have indefinite edges, interpatch distance at the peatland scale was measured as the average distance among the centers of sampled clusters (*D*_cl_; determined using GPS coordinates).

Considering the nested structure of habitat for *M. knabi* and previous documentation of cross-scale effects (Rasic and Keyghobadi 2012), we were also interested in whether the isolation of a study unit (i.e., each plant, cluster, and peatland) within its broader landscape context could influence genetic differentiation among sampled patches within that unit. We measured isolation of each plant within its respective cluster as the plant's distance to the cluster center (*I*_pl_). We measured isolation of each cluster within the peatland as the number of plants within a 10-m-wide buffer around the cluster (*I*_cl_) by analyzing maps of interpolated plant-count data collected in 2009–2010 (Rasic and Keyghobadi 2012) in ArcGIS version 9.3 (ESRI, Redlands, CA). Isolation of each peatland (*I*_ptld_) was measured as the distance from the center of the peatland to the center of the nearest neighboring peatland using Google Earth 6.2. Use of center-to-center peatland distances is justified by the small size of peatlands relative to the distances separating them (edge-to-edge distances are on average three times larger than patch radii) and a high correlation of center-to-center distances with edge-to-edge distances between peatlands (Pearson *r* = 0.974). Subsequently, models at the plant scale included isolation of each plant within its respective cluster (*I*_pl_), isolation of that cluster within its respective peatland (*I*_cl_), and isolation of that peatland (*I*_ptld_), as predictor variables. Likewise, cluster scale analyses included *I*_cl_ and *I*_ptld_ as predictors, and peatland scale analyses included *I*_ptld_ (Fig.2; Table S3).

### Amount of habitat

At the plant scale, the amount of habitat (A) in the local “landscape” (i.e., in each individual sampled plant) was quantified as the number of leaves per plant (*A*_pl_; Fig.2; Table S3). At the cluster and peatland scales, respectively, the amount of habitat was the number of plants per cluster (*A*_cl_) and the average plant density in the sampled peatland (from the 2009 to 2010 plant-count data; Rasic and Keyghobadi 2012) multiplied by peatland area (*A*_ptld_). Peatland area was measured in ArcGIS using a combination of high resolution enhanced Forest Resource Inventory aerial imagery (eFRI; Ontario Ministry of Natural Resources, 2006) and previously recorded GPS transect points (Rasic and Keyghobadi 2012). As for isolation, we were also interested in whether the amount of habitat beyond the scale of interest had an effect on genetic differentiation. Thus, at the plant scale, the amount of habitat in the surrounding cluster (*A*_cl_) and peatland (*A*_ptld_) were included in the model, while at the cluster scale, *A*_ptld_ was included (Fig.2; Table S3).

### Predictor variables at each scale

Separate data sets containing the response (*F*_ST_) and predictor variables were constructed for each spatial scale (Table S3). At the plant scale, eight independent predictors were considered to be important habitat structure metrics: mean patch size of the sampled leaves (*S*_lf_), mean pairwise distance among the sampled leaves (*D*_lf_), isolation of the plant (*I*_pl_), cluster (*I*_cl_), and peatland (*I*_ptld_), and the amount of habitat in the plant, cluster, and peatland (i.e., *A*_pl_, *A*_cl_, *A*_ptld_). The predictor variables *S*_lf_, *D*_lf_, and *A*_pl_ represent focal scale habitat metrics, whereas *I*_pl_, *A*_cl_, *I*_cl,_ and *A*_ptld_ represent habitat structure at broader scales. At the cluster scale, the six predictors included: mean patch size of sampled plants (*S*_pl_), mean pairwise distance among sampled plants (*D*_pl_), isolation of the cluster (*I*_cl_) and peatland (*I*_ptld_), and the amount of habitat in the cluster (*A*_cl_) and peatland (*A*_ptld_). Here, *S*_pl_, *D*_pl_, and *A*_cl_ are focal scale habitat metrics, while *I*_cl_, *I*_ptld_, and *A*_ptld_ represent broader scale habitat variables. At the peatland scale, predictor variables included: mean patch size of the sampled clusters (*S*_cl_), mean pairwise distance among sampled clusters (*D*_cl_), isolation of the peatland (*I*_ptld_), and the amount of habitat in the peatland (*A*_ptld_). As the effect of interpatch distance may depend on the amount of habitat in the landscape, interactions between mean pairwise distance among sampled patches and the amount of habitat at the focal scale were also included at each scale (plant scale, *D*_lf_:*A*_pl_; cluster scale, *D*_pl_:*A*_cl_; peatland scale, *D*_cl_:*A*_ptld_).

Pearson's pairwise correlation coefficient was assessed between all predictor variables within each data set to screen for high collinearity (*r* > 0.7; Dormann et al. 2013). As habitat metrics differ in units and scale, to aid in comparison of estimated coefficients, each predictor was standardized within each data set by subtracting the mean and dividing by the standard deviation (Tables S4–S6; Schielzeth 2010).

### Statistical modeling and multimodel inference

The influence of habitat structure on genetic differentiation (*F*_ST_) at each spatial scale was analyzed using generalized linear mixed models (GLMMs), fit using the lme4 package (Bates and Maechler 2010) in R (R Core Team 2013) and multimodel inference (Burnham and Anderson 2002). We accounted for potential covariance within nested spatial units by including a random intercept varying among systems, among peatlands within systems, and among clusters within peatlands.

At each scale, we generated a candidate set of models based on the additive combinations of predictors measured for that scale, all of which were expected to be potentially important, as well as an interaction term between interpatch distance and amount of habitat at the focal scale, which was also hypothesized to have a meaningful effect on genetic differentiation (Tables S3, S7). There were 512, 128, and 32 models, respectively, at the plant, cluster, and peatland scales. Models were ranked separately for each scale according to corrected Akaike information criterion values (AIC_c_; Akaike 1973; Sugiura 1978). Akaike model weights (*w*_*i*_) were calculated and interpreted as the probability that a model explains genetic differentiation, where a top-ranked model with AIC_c_ two units less than the second-ranked model and *w*_*i*_ > 0.9 was considered strong evidence in support of the best model (Burnham and Anderson 2002). When a single best model in the set was not clear, model averaging was conducted using all models in the set. Relative variable importance (*w*_+_[*i*]) was assessed for each predictor to identify the most important habitat metric at each scale by summing the Akaike weights of the target predictor across the models in which it was present. Model-averaged parameter estimates (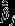) and their unconditional standard errors were calculated using the weighted average of the estimates from the models in which the target parameter was present. Model averaging and calculation of parameter estimates were conducted using MuMIn (Bartoń 2009) and AICcmodavg packages (Mazerolle 2012) in R (R Core Team 2013). Marginal *R*^2^_GLMM(*m*)_ and conditional *R*^2^_GLMM(*c*)_ was calculated following Nakagawa and Schielzeth (2013) when a single best model was apparent.

## Results

### Genetic diversity and structure

A total of 1199 individuals were genotyped from 195 leaves, 65 plants, 29 clusters, and seven peatlands. For modeling habitat amount and configuration effects on genetic differentiation, at least three individuals per leaf were required for plant scale analysis, such that the plant scale data set consisted of 921 individuals (average 4.7 individuals per leaf; Table S8). The cluster scale data set consisted of 1165 individuals (average 13.4 individuals per plant; Table S9), while all individuals were included in the peatland scale analysis (Table S10). One locus (mk80) was identified as potentially under positive selection and was removed from analyses. Based on 10 remaining microsatellites, the mean number of alleles (*N*_A_) at the plant scale ranged from 2.53 to 3.60, while the mean observed (*H*_O_) and expected heterozygosities (*H*_E_) ranged from 0.51 to 0.66 and 0.34 to 0.57, respectively (Table S8). At the cluster scale, *N*_A_ = 3.70–4.87, *H*_O_ = 0.48–0.64, and *H*_E_ = 0.50–0.55 (Table S9). At the peatland scale, *N*_A_ = 5.62–6.56, *H*_O_ = 0.55–0.59, and *H*_E_ = 0.53–0.56 (Table S10). The average number of alleles across loci and peatlands was 7.77 (SE = 0.99) in System 1 and 9.27 (SE = 0.99) in System 2. Observed and expected heterozygosity were not significantly different between the systems (System 1: *H*_O_ = 0.60 [SE = 0.04], *H*_*E*_ = 0.56 [SE = 0.04]; System 2: *H*_O_ = 0.56 [SE = 0.03], *H*_E_ = 0.55 [SE = 0.03]).

**Figure 3:**
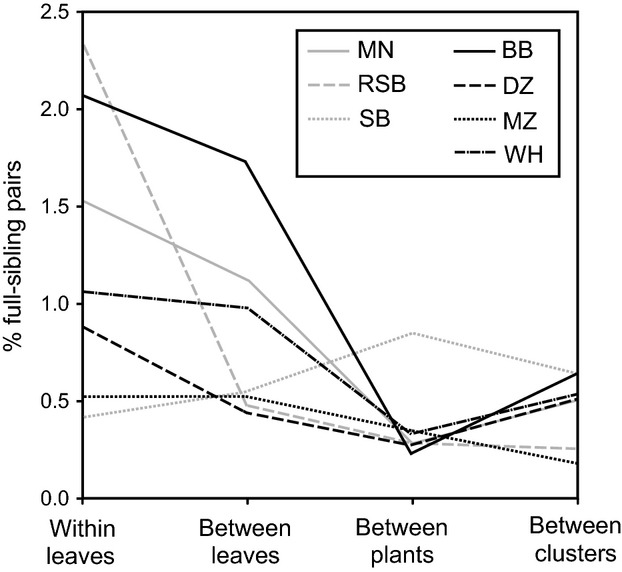
Percentage of full-sibling relationships measured among individuals from within the same leaf, between leaves within plants, between plants within clusters, and between clusters within peatlands, shown separately for each peatland.

The incidence of full-sibling pairs was highest among individuals collected from the same leaf (0.52–2.38%) and decreased steadily in between-leaf and between-plant comparisons, but then increased slightly in between-cluster comparisons in all peatlands except SB (Fig.3). In SB, the percentage of full-sibling relationships was lowest among individuals sampled from the same leaf (0.42%) and highest among individuals from different plants (0.64%; Fig.3).

The hierarchical AMOVA indicated that the highest level of variance occurs within individuals and at the leaf scale, in both peatland systems (Table1). Hierarchical *F*-statistics in both systems were significant (*P *<* *0.01) at all spatial scales, including within individuals. Values at the individual level are equivalent to inbreeding coefficients, and similar to Rasic and Keyghobadi (2012), we observed negative values implying individuals are highly outbred (Table2).

**Table 1:** Summary of hierarchical analysis of variance components in System 1 and System 2. System 2 values are the average variance components measured after one peatland at a time was left out of the analysis

System	Peatland	Cluster	Plant	Leaf	Individual	Error
1	0.0922	0.0201	0.0180	0.1276	−0.4442	6.1029
2	0.0629	0.0269	0.0524	0.1739	−0.4550	5.9375

**Table 2:** Matrix of hierarchical *F*-statistics among peatlands, clusters, plants, and leaves within System 1 (SYS1) and System 2 (SYS2). Values represent *F*_ST_ values among the “column” scale within the “row” scale. Statistical significance was obtained by permuting whole units of the lower scale within units of the scale of interest, while maintaining the nested structure within broader scales. For example, in the plant column, whole units of the leaf were permutated among plants, but retained within respective clusters. System 2 values are the average *F*-statistics after removing one peatland at a time from the analysis (all values *P *<* *0.01)

	Peatland		Cluster		Plant		Leaf		Individual	
	SYS1	SYS2	SYS1	SYS2	SYS1	SYS2	SYS1	SYS2	SYS1	SYS2	
Total	0.0156	0.0108								
Peatland			0.0034	0.0047						
Cluster					0.0031	0.0091				
Plant							0.0220	0.0307		
Leaf									−0.0785	−0.0830

At the plant scale, *F*_ST_ values computed among the three leaves within each plant ranged from −0.0037 to 0.1307, and 21.5% of the values were significantly >0 (*P *<* *0.05; Table S8). At the cluster scale, *F*_ST_ measured among the three plants within each cluster ranged from −0.0003 to 0.0871, and 37.9% of values were significant, whereas *F*_ST_ computed among clusters within peatlands ranged from 0.0037 to 0.0169, and 85.7% were significant (Tables S9, S10).

### Model selection

There were no strong correlations between predictor variables in any of the data sets (Tables S11–S13). Within the plant scale model set, no single model had a high probability of being the “best”, as eight models were within ΔAIC_c_ < 2 and Akaike weight (*w*_*i*_) ranged from 0.063 to 0.025 (Table S14). The cumulative sum of the Akaike model weights (0.339) among the top eight models suggests considerable model uncertainty. Nonetheless, all models within ΔAIC_c_ < 2 contained predictors for the isolation of the peatland in the surrounding system (*I*_ptld_), and model averaging indicated that *I*_ptld_ had the highest relative importance with a model-averaged weight (*w*_+_[*i*]) of 0.916 (Table3). Peatland isolation had a positive effect on genetic differentiation among leaves (Table3).

**Table 3:** Model-averaged Akaike weights (*w*_+_[*i*]), parameter estimates (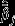), and standard errors (SE) for effects of metrics of habitat configuration (patch size, interpatch distance, and patch isolation) and amount of habitat on genetic differentiation (*F*_*ST*_) at the plant, cluster, and peatland scales. Colons indicate interaction terms

	Patch size				Interpatch distance and patch isolation				Amount of habitat			
Scale	Parameter	*w*_+_(*i*)	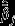	SE	Parameter	*w*_+_(*i*)	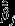	SE	Parameter	*w*_+_(*i*)	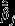	SE
Plant	*S*_lf_	0.246	−0.0042	0.005	*D*_lf_	0.369	−0.0039	0.0050	*A*_pl_	0.227	−0.0004	0.005
					*I*_pl_	0.409	−0.0063	0.0047	*A*_cl_	0.274	−0.0075	0.005
					*I*_cl_	0.592	−0.0103	0.0054	*A*_ptld_	0.188	−0.0027	0.006
					*I*_ptld_	0.916	0.0132	0.0048	*D*_lf_:*A*_pl_	0.019	−0.0002	0.002
Cluster	*S*_pl_	0.483	0.0059	0.004	*D*_pl_	0.150	0.0000	0.0035	*A*_cl_	0.169	−0.0003	0.004
					*I*_cl_	0.170	−0.0009	0.0036	*A*_ptld_	0.585	0.0061	0.005
					*I*_ptld_	0.910	0.0093	0.0036	*D*_pl_:*A*_cl_	0.009	0.0097	0.005
Peatland	*S*_cl_	0.996	−0.0015	0.000	*D*_cl_	1.000	−0.0037	0.0017	*A*_ptld_	1.000	−0.0083	0.003
					*I*_ptld_	0.004	0.0011	0.0008	*D*_cl_:*A*_ptld_	1.000	−0.0101	0.003

At the cluster scale, three models were within ΔAIC_c_ < 2 and had a cumulative *w*_*i*_ = 0.501 (Table S14). All models within ΔAIC_c_ < 2 contained isolation of the peatland (*I*_ptld_), which was assigned high relative importance (0.910) following model averaging (Table3). The effect of peatland isolation (*I*_ptld_) on *F*_ST_ at the cluster scale was positive.

**Figure 4:**
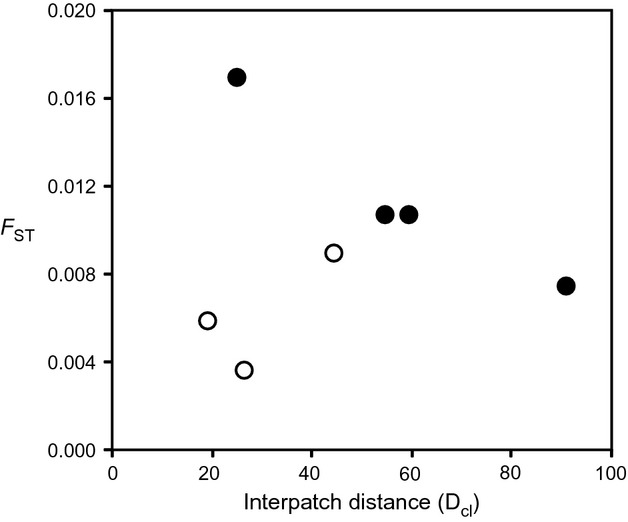
Interaction of interpatch distance and habitat amount at peatland scale. The relationship between genetic differentiation (*F*_ST_) among clusters and interpatch distance (*D*_cl_) is shown when the amount of habitat in the surrounding peatland (*A*_ptld_) is high (filled circles) and low (open circles).

At the peatland scale, a model including mean size of sampled clusters (*S*_cl_), mean interpatch distance among sampled clusters (*D*_cl_), amount of habitat in the surrounding peatland (*A*_ptld_), and an interaction between interpatch distance and amount of habitat in the peatland (*D*_cl_:*A*_ptld_) was clearly identified as the best model (*w*_*i*_ = 0.994; Table S14; *R*^2^_GLMM(*m*)_ = 0.246, *R*^2^_GLMM(*c*)_ = 0.999). The second-ranked model had ΔAIC_c_ = 11.14. All of the predictors in the top model (*S*_cl_, *D*_cl_, *A*_ptld_, and *D*_cl_:*A*_ptld_) had equally high importance (0.996–1.000), and their estimated effects on *F*_ST_ among clusters were all negative. Post hoc examination of the interaction between interpatch distance (*D*_cl_) and amount of habitat in the peatland (*A*_ptld_) was conducted by dividing the data into high and low *A*_ptld_ groups according to positive and negative standardized *A*_ptld_ values, respectively, and assessing the relationship between *F*_ST_ and interpatch distance (*D*_cl_) for each group. Genetic differentiation increased with interpatch distance when the amount of surrounding habitat was low (Pearson *R*^2^ = 0.581), but decreased with interpatch distance when the amount of surrounding habitat was high (Pearson *R*^2^ = 0.938; Fig.4).

## Discussion

Our study indicates that aspects of both habitat amount and configuration affect genetic differentiation of *M. knabi*; however, the relative importance of the amount of habitat in the landscape versus its spatial configuration is scale dependent, and some cross-scale effects are apparent.

### Processes determining genetic differentiation at different spatial scales

The transition from fine (leaf) to broad (peatland) spatial scales involves a shift from predominantly individual to population level processes (Krawchuk and Taylor 2003). Consistent with previous work on this system (Rasic and Keyghobadi 2012), we detected significant genetic structure among larvae sampled from different leaves within pitcher plants, despite the small average distance among leaves (10.55 cm; Table S3) which is within the expected dispersal range of adult *M. knabi* (Krawchuk and Taylor 2003). Larvae colonize *S. purpurea* through oviposition. Female oviposition decisions, particularly the spatial distribution of eggs (e.g., clumped within, versus spread among, leaves and plants), should be an important determinant of observed spatial genetic structure among larvae at a fine scale (Anderson and Dunham 2008; Goldberg and Waits 2010). Here, the situation is likely similar to that of metapopulation genetics, where if colonization of each empty patch is dominated by one or a few individuals, analogous to one or two females clustering their eggs within a leaf, then high genetic differentiation among patches will result (Hanski and Gaggiotti 2004). In this study, individuals sampled from the same leaf were 1.5 times more likely to be full siblings than individuals sampled from different leaves and three times more likely to be full siblings than individuals sampled from different plants and clusters (Fig.3). This result confirms the importance of oviposition as a key process affecting genetic differentiation among larval samples collected at the finest scale.

At the broadest scale of this study (among clusters within peatlands), samples were highly differentiated with the greatest proportion of significant *F*_ST_ values (85.71%) and second highest hierarchical *F-*statistic values (Table2). Previous work indicates that *M. knabi* dispersal is likely limited at this scale (Krawchuk and Taylor 2003; Rasic and Keyghobadi 2012). As such, neutral genetic differentiation is expected to be influenced by genetic drift acting on partially or completely isolated populations.

The intermediate cluster scale likely contains a combination of fine- and broad-scale processes that interact to determine genetic structure among larvae sampled from different plants within a cluster. As fine-scale processes (primarily oviposition) are scaled up and broader scale processes (gene flow-drift) are scaled down, we might expect the magnitude of the effects of both sets of processes on genetic differentiation to attenuate. Consistent with this expectation, we observed on average the lowest genetic variance components and hierarchical *F*-statistic values at the cluster scale in both peatland systems (Tables1,2). Overall, while *M. knabi* exhibits genetic structure at all three sampling levels, patterns of genetic differentiation appear to be driven by processes operating at two key domains of scale: oviposition at a fine scale and gene flow-drift at a broader scale.

### Effects of habitat amount and configuration at different scales

Given that female oviposition and potentially differential larval survival are expected to be dominant processes determining genetic differentiation at the plant scale, we might expect patch (i.e., leaf) size to be an important predictor of genetic differentiation at this scale. Leaf size is positively correlated with larval abundance (Krawchuk and Taylor 2003), influences accessibility by females (Trzcinski et al. 2003), and affects capture rate of insect prey, an important resource for developing larvae (Wolf 1981; Cresswell 1993; Heard 1998). However, multimodel inference identified a broad-scale variable, peatland isolation (*I*_ptld_), as the only important predictor of *F*_ST_ among leaves (Table3). This result is consistent with previous work that found genetic distances among individuals of *M. knabi* at a fine spatial scale are influenced by the broad-scale isolation and density of pitcher plants (Rasic and Keyghobadi 2012). Given the strict habitat requirement for developing larvae, it is likely that female oviposition behavior will respond to habitat structure at more than one spatial scale, and it has been suggested that low availability of oviposition sites at a broader scale (reflected in low density or high isolation of pitcher plants) may make females “choosy” and more likely to aggregate eggs within single leaves (Rasic and Keyghobadi 2012). Our observation of a positive relationship between *F*_ST_ and peatland isolation supports this hypothesis. Interestingly, Spruce Bog was the only site in which we found fewer full-sibling pairs within leaves than between leaves, plants, or clusters (Fig.3). This peatland is very small, with an unusually high density of pitcher plants. The wider spatial dispersion of sibling larvae observed here relative to other studied peatlands also supports the hypothesis that adult females aggregate or disperse eggs in response to availability of oviposition sites perceived at broader scales.

At the peatland scale, where drift and gene flow are dominant processes, we would expect size and distance among sampled patches at the focal scale to determine levels of genetic differentiation. Low effective population size should increase the effect of drift in small patches (i.e., clusters), while gene flow should decrease with increased patch distances. We did indeed find that patch size (*S*_cl_) and interpatch distance (*D*_cl_) were important predictors of genetic differentiation among clusters and that higher differentiation, which results from higher levels of drift, was associated with smaller patch size (Table3). Similar patterns have previously been described for numerous species in fragmented landscapes (Frankham 1997; Holmes et al. 2013). However, we also found the amount of habitat surrounding the patch (*A*_ptld_) and its interaction with interpatch distance (*D*_cl_:*A*_ptld_) were important (Table3). This result is consistent with ecological studies that have suggested the influence of habitat configuration depends on the amount of habitat in the surrounding landscape and is most important when the amount of habitat is generally low (e.g., Fahrig 1997; Trzcinski et al. 1999; Smith et al. 2011).

For genetic data, we would expect a stronger positive effect of interpatch distance on genetic differentiation when the amount of habitat in the landscape is low; with increasing habitat amount, unsampled intervening patches that can act as stepping-stones for gene flow, and a larger regional effective population size, may dampen such a relationship. Consistent with this expectation, we did see greater genetic differentiation with increasing pairwise patch distance when the amount of habitat in the landscape was low (Fig.4). However, we also found an unexpected trend of lower differentiation with increasing interpatch distance when habitat amount was high. Overall, our observed interaction between interpatch distance and habitat amount (*D*_cl_:*A*_ptld_) must be interpreted cautiously given a limited number of data points, and considering that peatlands with low habitat amount were all from System 1 and those with high habitat amount were all from System 2. Further study is needed to determine whether the observed pattern is an artifact of sampling design and to establish more solidly the nature of interpatch distance and habitat amount interactions. Nonetheless, studies examining patch size and distance effects on the genetics of fragmented populations would likely benefit by also considering the amount of habitat in the landscape.

At the intermediate cluster scale, low importance was assigned to focal scale patch size (*S*_pl_) and interpatch distance (*D*_pl_), which is consistent with the expectations that gene flow within clusters is not limited (Rasic and Keyghobadi 2012), and that the influence of gene flow and drift as underlying processes are attenuated at this scale. The broad-scale variable of peatland isolation (*I*_ptld_) was the only variable identified as highly important, and the weight assigned to it, as well as the direction of its effect, was similar at the plant and cluster scales (Table3). This suggests that fine-scale oviposition continues to be an important process influencing genetic differentiation at the cluster scale.

### Importance of habitat amount versus configuration for genetic differentiation

Simulation modeling experiments to quantify the relative influence of habitat amount and configuration on genetic structure conclude that habitat configuration is more important than habitat amount in predicting genetic differentiation and that patch characteristics are among the strongest individual predictors of genetic structure (Bruggeman et al. 2010; Cushman et al. 2012). Our empirical study complements and adds to these findings and suggests that both habitat amount and configuration can be important determinants of genetic differentiation in natural systems, and that their relative importance is scale dependent. Furthermore, at a scale where gene flow and genetic drift are the primary drivers of neutral differentiation, our data suggested an interaction between interpatch distance and habitat amount on genetic differentiation, consistent with previous ecological work suggesting that the effects of habitat configuration are likely to depend on the amount of habitat in the landscape. Another key finding of our study are cross-scale effects, such as at the plant scale where isolation of the peatland in the broader landscape is a strong predictor of genetic differentiation measured at fine scales. These latter two findings indicate that in landscape genetic and habitat fragmentation studies, habitat structure beyond the scale of sampling may be important and should be considered when investigating patterns of genetic differentiation.

Control of habitat amount and configuration in natural systems is extremely challenging, and experimental manipulations are generally impractical for genetic studies because of the time lags required for genetic structure to respond to changes in landscape conditions (Landguth et al. 2010). Our study took advantage of a microcosm system in which habitat amount and configuration vary naturally, to assess their relative effects on levels of genetic differentiation. We thus provide a framework through which the importance of different components of habitat structure to genetic patterns in other natural systems can be examined.

## Data Accessibility

Genotype data available from the Dryad Digital Repository: http://doi.org/10.5061/dryad.79gk4

## Supporting Information

**Figure S1.**Measurements of pitcher plant leaves: 1 = pitcher mouth, 2 = pitcher width, 3 = hood height, and 4 = pitcher height. **Table S1.** Names of sampled peatlands, with area (m^2^) and UTM coordinates (zone 17) of sampled clusters. **Table S2.** Summary statistics of the correlation between leaf measurements (1 = pitcher mouth, 2 = pitcher width, 3 = hood height, 4 = pitcher height) and potential leaf volume (mL). **Table S3.** Predictor variables included in models of genetic differentiation at plant, cluster, and peatland scales. **Table S4.** Plant scale habitat metrics, measured for each plant (Pl) in each cluster (Cl), peatland (Ptld), and system (Sys). **Table S5.** Cluster scale habitat metrics measured for each cluster (Cl), in each peatland (Ptld), and system (Sys). **Table S6.** Peatland scale habitat metrics measured for each peatland (Ptld), in each system (Sys). **Table S7.** Global models used to generate candidate model sets in the plant, cluster, and peatland scale datasets. **Table S8.** Summary of plant scale genetic data with the average number of alleles (*N*_A_), observed (*H*_O_) and expected (*H*_E_) levels of heterozygosity, inbreeding coefficient (*F*_IS_), and *F*_ST_ values (bolded values *P *<* *0.05). **Table S9.** Summary of cluster scale genetic data with the average number of alleles (*N*_A_), observed (*H*_O_) and expected (*H*_E_) levels of heterozygosity, inbreeding coefficient (*F*_IS_), and *F*_ST_ values (bolded values *P *<* *0.05). **Table S10.** Summary of peatland scale genetic data with the average number of alleles (*N*_A_), observed (*H*_O_) and expected (*H*_E_) levels of heterozygosity, inbreeding coefficient (*F*_IS_), and *F*_ST_ values (bolded values *P *<* *0.05). **Table S11.** Pearson correlation coefficients indicating the correlation between predictor variables used in plant scale models (significance levels **P *<* *0.05, ** <0.01, *** <0.001). **Table S12.** Pearson correlation coefficients indicating the correlation between predictor variables used in cluster scale models (significance levels **P *<* *0.05, ** <0.01, *** <0.001). **Table S13.** Pearson correlation coefficients indicating the correlation between predictor variables used in peatland scale models (significance levels **P *<* *0.05, ** <0.01, *** <0.001). **Table S14.** Summary of model selection statistics for candidate models at the plant, cluster, and peatland scales, with log likelihood (logLik) statistics, corrected Akaike information criterion (AICc) Δ*i* AICc, and Akaike weights (*w*_*i*_).
