# Supplementary material for: The relative influence of habitat amount and configuration on genetic structure across multiple spatial scales
Source: Ecol Evol. 2014 Dec 5;5(1):73–86. doi: 10.1002/ece3.1325 (PMC4298435; doi:10.1002/ece3.1325)
Supplement: Supplementary file 1 — Figure S1.Measurements of pitcher plant leaves: 1 = pitcher mouth, 2 = pitcher width, 3 = hood height, and 4 = pitcher height. Table S1. Names of sampled peatlands, with area (m2) and UTM coordinates (zone 17) of sampled clusters. Table S2. Summary statistics of the correlation between leaf measurements (1 = pitcher mouth, 2 = pitcher width, 3 = hood height, 4 = pitcher height) and potential leaf volume (mL). Table S3. Predictor variables included in models of genetic differentiation at plant, cluster, and peatland scales. Table S4. Plant scale habitat metrics, measured for each plant (Pl) in each cluster (Cl), peatland (Ptld), and system (Sys). Table S5. Cluster scale habitat metrics measured for each cluster (Cl), in each peatland (Ptld), and system (Sys). Table S6. Peatland scale habitat metrics measured for each peatland (Ptld), in each system (Sys). Table S7. Global models used to generate candidate model sets in the plant, cluster, and peatland scale datasets. Table S8. Summary of plant scale genetic data with the average number of alleles (NA), observed (HO) and expected (HE) levels of heterozygosity, inbreeding coefficient (FIS), and FST values (bolded values P < 0.05). Table S9. Summary of cluster scale genetic data with the average number of alleles (NA), observed (HO) and expected (HE) levels of heterozygosity, inbreeding coefficient (FIS), and FST values (bolded values P < 0.05). Table S10. Summary of peatland scale genetic data with the average number of alleles (NA), observed (HO) and expected (HE) levels of heterozygosity, inbreeding coefficient (FIS), and FST values (bolded values P < 0.05). Table S11. Pearson correlation coefficients indicating the correlation between predictor variables used in plant scale models (significance levels *P < 0.05, ** <0.01, *** <0.001). Table S12. Pearson correlation coefficients indicating the correlation between predictor variables used in cluster scale models (significance levels *P < 0.05, ** <0.01, *** <0.001). T [file ece30005-0073-sd1.pdf]

Table S1. Names of sampled peatlands, with area (m^2) and UTM coordinates (zone 17) of sampled clusters.

System	Peatland name	Code	Area (m^2)	Cluster	Easting	Northing
1	Minor Lake	MN	8,294	1	701470.35	5057404.66
				2	701431.21	5057416.08
				3	701492.58	5057439.70
				4	701455.00	5057477.32
				5	701406.24	5057471.99
1	'Roadside'	RSB	3,395	6	705952.47	5051985.57
				7	705946.24	5051996.82
				8	705930.78	5052027.05
1	Spruce	SB	1,895	9	705175.87	5052062.63
				10	705198.36	5052047.58
				11	705187.01	5052024.75
2	'Buggy'	BB	34,532	12	679543.08	5049240.78
				13	679534.18	5049207.55
				14	679435.99	5049239.82
				15	679475.67	5049063.61
				16	679535.96	5049087.45
2	Dizzy Lake	DZ	36,143	17	680239.54	5046841.01
				18	680367.22	5047086.61
				19	680426.09	5047190.66
				20	680382.56	5047150.68
				21	680320.50	5046946.62
2	Mizzy Lake	MZ	80,772	22	681075.61	5047398.62
				23	680978.63	5047381.92
				24	680940.23	5047357.64
2	Wolf Howl	WH	24,003	25	680310.84	5049817.92
				26	680347.46	5049867.28
				27	680285.56	5049907.96
				28	680247.52	5049928.95
				29	680233.54	5049869.09

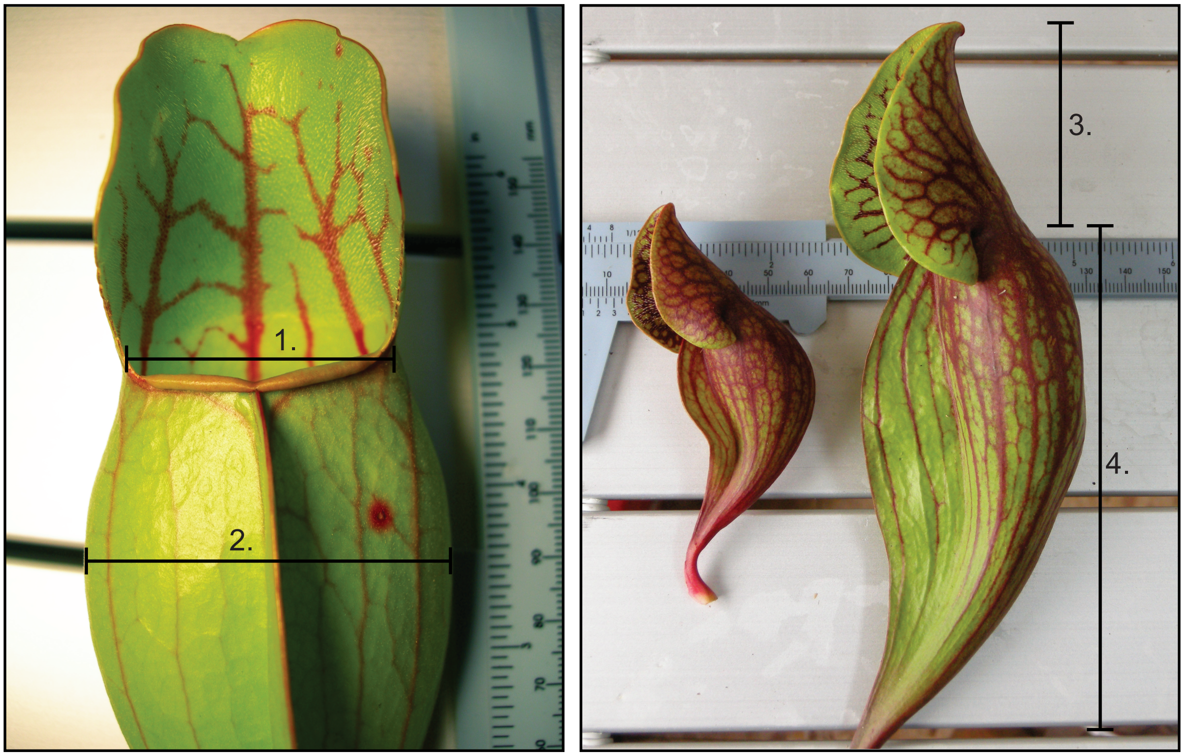

Figure S1. Measurements of pitcher plant leaves: 1 = pitcher mouth, 2 = pitcher width, 3 = hood height, and 4 = pitcher height.

Table S2. Summary statistics of the correlation between leaf measurements (1 = pitcher mouth, 2 = pitcher width, 3 = hood height, 4 = pitcher height) and potential leaf volume (mL). Potential leaf volume was measured for 249 leaves by emptying the leaf contents and filling the leaf with water to volumetric capacity. Water was then poured into a graduated cylinder and measured. Potential leaf volume was cube-root transformed for correlation analysis.

Leaf measurement	R^2	r	p-value	df
1	0.7555	0.8612	< 0.0001	246
2	0.9169	0.9575	< 0.0001	246
3	0.6838	0.8269	< 0.0001	246
4	0.0015	0.8727	< 0.0001	239

Table S3. Predictor variables included in models of genetic differentiation at plant, cluster, and peatland scales. Descriptions of measures of patch size (S), inter-patch distance (D), patch isolation (I), and amount of habitat (A) at each scale are given with the mean and range of observed values.

Code	Description	Measurement	Mean	Range
Plant scale				
S_{lf}	Patch size of leaf	Average size of the three sampled leaves (mm)	32.17	24.33 – 44.67
D_{lf}	Inter-patch distance of leaf	Average pairwise distance among the three sampled leaves (cm)	10.55	4.17 – 21.00
I_{pl}	Isolation of plant	Distance of plant to centre of cluster (m)	3.45	0.51 – 8.28
I_{cl}	Isolation of cluster	Number of plants in 10 m-wide buffer around sampled cluster	15.60	0 – 53
I_{ptld}	Isolation of peatland	Distance to nearest peatland (m)	260.17	131.39 – 531.60
A_{pl}	Amount of habitat in plant	Number of leaves per plant	7.88	4 – 17
A_{cl}	Amount of habitat in cluster	Number of plants per cluster	113.91	15 – 346
A_{ptld}	Amount of habitat in peatland	Average density of plants × peatland area (m^2)	37,791.15	2,993.11 – 120,547.80
Cluster scale				
S_{pl}	Patch size of plant	Average number of leaves per plant for the three sampled plants	7.96	4.67 – 11.33
D_{pl}	Inter-patch distance of plant	Average pairwise distance among the three sampled plants (m)	4.33	1.82 – 7.32
I_{cl}	Isolation of cluster	Number of plants in 10 m-wide buffer around sampled cluster	16.52	0 – 53
I_{ptld}	Isolation of peatland	Distance to nearest peatland (m)	259.91	131.39 – 531.60
A_{cl}	Amount of habitat in cluster	Number of plants per cluster	116.55	15 – 346
A_{ptld}	Amount of habitat in peatland	Average density of plants × peatland area (m^2)	42,075.03	2,993.11 – 120,547.80
Peatland scale				
S_{cl}	Patch size of cluster	Average number of plants in the sampled clusters	122.95	46.99 – 217.77
D_{cl}	Inter-patch distance of cluster	Average pairwise distance among the sampled clusters (m)	45.99	19.53 – 91.14
I_{ptld}	Isolation of peatland	Distance to nearest peatland (m)	262.64	131.52 – 532.11
A_{ptld}	Amount of habitat in peatland	Average density of plants × peatland area (m^2)	42,928.81	2,993.11 – 120,547.80

Table S4. Plant scale habitat metrics, measured for each plant (Pl) in each cluster (Cl), peatland (Ptld), and system (Sys). Values were standardized by subtracting the mean and dividing by the standard deviation. Codes correspond to those in Table S3: S_{lf} = patch size of the leaf, D_{lf} = inter-patch distance among leaves, A_{pl} = amount of habitat in the plant, I_{pl} = isolation of the plant, A_{cl} = amount of habitat in the cluster, I_{cl} = isolation of the cluster, A_{ptld} = amount of habitat in the peatland, and I_{ptld} = isolation of the peatland.

Sys	Ptld	Cl	Pl	S_{lf}	D_{lf}	A_{pl}	I_{pl}	A_{cl}	I_{cl}	A_{ptld}	I_{ptld}
1	MN	1	1	-1.4652	-0.9595	-0.6142	0.539	-0.1159	-0.1281	-0.8197	-0.2876
1	MN	1	2	-0.5363	-1.4320	0.3675	-0.934	-0.1159	-0.1281	-0.8197	-0.2876
1	MN	1	3	-1.6796	-1.4320	1.6764	0.077	-0.1159	-0.1281	-0.8197	-0.2876
1	MN	2	4	0.4638	0.2864	-0.9414	0.417	0.0362	0.1120	-0.8197	-0.2876
1	MN	3	5	-1.3936	0.2005	-0.2870	1.331	-0.6540	-0.2881	-0.8197	-0.2876
1	MN	3	6	-0.9650	-1.6039	-0.2870	-0.405	-0.6540	-0.2881	-0.8197	-0.2876
1	MN	3	7	-0.3220	-0.4009	-0.6142	0.635	-0.6540	-0.2881	-0.8197	-0.2876
1	MN	4	8	-0.3936	-0.1432	-0.6142	-1.916	-1.0050	-0.1281	-0.8197	-0.2876
1	MN	4	9	-1.3936	0.2864	-0.9414	0.967	-1.0050	-0.1281	-0.8197	-0.2876
1	MN	4	10	2.1071	1.7471	-0.2870	1.529	-1.0050	-0.1281	-0.8197	-0.2876
1	MN	5	11	-1.1793	-1.3462	0.0403	0.534	-0.2563	0.2721	-0.8197	-0.2876
1	MN	5	12	-0.7506	-1.0884	-0.2870	-1.356	-0.2563	0.2721	-0.8197	-0.2876
1	RSB	6	13	-1.6796	-1.0024	-0.9414	1.507	-0.9465	0.1120	-0.7347	1.2384
1	RSB	6	14	-1.1793	-0.2291	-1.2687	0.459	-0.9465	0.1120	-0.7347	1.2384
1	RSB	7	15	-0.4649	0.5012	-0.9414	-0.253	-0.5488	2.2730	-0.7347	1.2384
1	RSB	7	16	0.3210	-0.2291	-0.6142	-0.438	-0.5488	2.2730	-0.7347	1.2384
1	RSB	7	17	0.5354	-0.4869	-0.9414	0.814	-0.5488	2.2730	-0.7347	1.2384
1	RSB	8	18	-0.2506	-0.8306	1.6764	-1.614	-0.8529	-0.4482	-0.7347	1.2384
1	RSB	8	19	-0.6079	-0.4009	0.3675	0.162	-0.8529	-0.4482	-0.7347	1.2384
1	RSB	8	20	1.1068	-0.2721	-0.6142	0.204	-0.8529	-0.4482	-0.7347	1.2384
1	SB	9	21	0.3924	-0.1174	-0.2870	-0.983	1.1476	-0.3682	-1.2679	-0.1032
1	SB	9	22	1.6070	2.3056	-1.2687	0.422	1.1476	-0.3682	-1.2679	-0.1032

1	SB	10	23	0.1067	2.6923	-1.2687	-1.218	2.7152	-0.3682	-1.2679	-0.1032
1	SB	10	24	-0.2506	0.2864	1.3492	1.507	2.7152	-0.3682	-1.2679	-0.1032
1	SB	10	25	1.0354	0.8020	1.0220	1.132	2.7152	-0.3682	-1.2679	-0.1032
1	SB	11	26	-1.3223	-0.3150	-1.2687	-0.442	-0.2212	-0.9284	-1.2679	-0.1032
1	SB	11	27	-0.8222	-0.1432	-0.9414	1.289	-0.2212	-0.9284	-1.2679	-0.1032
2	BB	12	28	-0.0363	0.1146	0.0403	-0.424	-0.7476	-0.2081	0.5074	1.7838
2	BB	12	29	-0.4649	-0.0573	0.6947	1.324	-0.7476	-0.2081	0.5074	1.7838
2	BB	13	30	0.0351	0.9737	0.3675	-1.351	-0.7242	-0.6883	0.5074	1.7838
2	BB	13	31	0.8211	0.5443	0.0403	-1.313	-0.7242	-0.6883	0.5074	1.7838
2	BB	13	32	-0.6793	-0.4009	-0.6142	1.149	-0.7242	-0.6883	0.5074	1.7838
2	BB	14	33	-0.5363	-0.5729	-0.6142	-0.770	0.3637	1.2325	0.5074	1.7838
2	BB	14	34	-0.3936	0.2865	0.3675	0.457	0.3637	1.2325	0.5074	1.7838
2	BB	15	35	0.6067	0.0288	-1.2687	-1.002	0.0830	2.9933	0.5074	1.7838
2	BB	15	36	1.3211	-0.1432	-0.6142	-1.420	0.0830	2.9933	0.5074	1.7838
2	BB	15	37	-1.1079	-0.4440	-1.2687	-0.774	0.0830	2.9933	0.5074	1.7838
2	DZ	16	38	-0.8222	-0.0573	0.3675	0.569	0.2585	-0.6083	0.2378	-0.8380
2	DZ	16	39	2.6787	1.1457	-0.6142	-0.321	0.2585	-0.6083	0.2378	-0.8380
2	DZ	16	40	1.3211	1.0598	0.0403	-1.039	0.2585	-0.6083	0.2378	-0.8380
2	DZ	17	41	0.2494	-0.7446	0.3675	0.144	-0.1978	-0.6883	0.2378	-0.8380
2	DZ	17	42	0.1067	0.4582	0.0403	-0.603	-0.1978	-0.6883	0.2378	-0.8380
2	DZ	18	43	0.4638	-0.0573	2.0037	0.672	-0.6307	-1.2485	0.2378	-0.8380
2	DZ	18	44	-0.2506	-0.2290	-0.2870	-0.864	-0.6307	-1.2485	0.2378	-0.8380
2	DZ	18	45	-0.1792	-0.4868	-0.6142	-0.365	-0.6307	-1.2485	0.2378	-0.8380
2	DZ	19	46	0.3924	1.6612	-0.9414	-0.142	-0.6073	-1.1685	0.2378	-0.8380
2	DZ	19	47	0.7497	0.8449	0.6947	0.083	-0.6073	-1.1685	0.2378	-0.8380
2	DZ	19	48	1.0354	1.9190	-1.2687	-0.051	-0.6073	-1.1685	0.2378	-0.8380
2	MZ	20	49	0.6781	1.4034	0.0403	-0.586	-1.1571	-0.5282	3.0153	-0.8380
2	MZ	20	50	1.1784	0.6301	2.6581	0.860	-1.1571	-0.5282	3.0153	-0.8380
2	MZ	20	51	0.5354	-0.4868	0.3675	-0.017	-1.1571	-0.5282	3.0153	-0.8380
2	MZ	21	52	2.1071	2.3495	-0.2870	0.339	-0.7944	-0.4482	3.0153	-0.8380
2	WH	22	53	-1.6080	-0.4868	2.3309	-0.395	1.5336	0.9124	0.3722	-0.8463
2	WH	22	54	-0.8222	-0.7446	-0.2870	0.182	1.5336	0.9124	0.3722	-0.8463
2	WH	23	55	-0.1076	-0.3151	1.0220	-1.763	0.6562	-0.2081	0.3722	-0.8463
2	WH	23	56	0.1781	-1.6468	1.6764	-0.650	0.6562	-0.2081	0.3722	-0.8463

2	WH	23	57	0.6067	2.0909	-0.2870	1.146	0.6562	-0.2081	0.3722	-0.8463
2	WH	24	58	-0.0363	-0.8307	2.9854	-1.741	0.2585	-0.2081	0.3722	-0.8463
2	WH	24	59	0.8924	-0.9165	0.0403	3.154	0.2585	-0.2081	0.3722	-0.8463
2	WH	24	60	-0.8936	-1.1312	-0.9414	0.809	0.2585	-0.2081	0.3722	-0.8463
2	WH	25	61	-1.2509	-1.3462	0.3675	0.297	-0.0925	0.4322	0.3722	-0.8463
2	WH	25	62	-0.8936	-1.2601	-0.2870	-0.550	-0.0925	0.4322	0.3722	-0.8463
2	WH	26	63	0.2494	0.0288	0.3675	0.138	2.1887	0.3521	0.3722	-0.8463
2	WH	26	64	1.5354	0.0288	1.0220	1.655	2.1887	0.3521	0.3722	-0.8463
2	WH	26	65	1.3211	0.1146	1.0220	-0.807	2.1887	0.3521	0.3722	-0.8463

Table S5. Cluster scale habitat metrics measured for each cluster (Cl), in each peatland (Ptld), and system (Sys). Values were standardized by subtracting the mean and dividing by the standard deviation. Codes correspond to those in Table S3: S_{pl} = patch size of the plant, D_{pl} = inter-patch distance among plants, A_{cl} = amount of habitat in the cluster, I_{cl} = isolation of the cluster, A_{ptld} = amount of habitat in the peatland, and I_{ptld} = isolation of the peatland.

Sys	Ptld	Cl	S_{pl}	D_{pl}	A_{cl}	I_{cl}	A_{ptld}	I_{ptld}
1	MN	1	0.7371	0.9261	-0.1444	-0.2092	-0.8454	-0.2804
1	MN	2	-1.2387	0.3195	0.0052	0.0401	-0.8454	-0.2804
1	MN	3	-0.6998	-0.0491	-0.6736	-0.3755	-0.8454	-0.2804
1	MN	4	-1.0593	0.1913	-1.0187	-0.2092	-0.8454	-0.2804
1	MN	5	-0.1609	-0.0411	-0.2825	0.2064	-0.8454	-0.2804
1	RSB	6	-1.5982	0.8914	-0.9612	0.0401	-0.7718	1.2161
1	RSB	7	-1.4186	-0.3747	-0.5701	2.2844	-0.7718	1.2161
1	RSB	8	0.7371	-1.4914	-0.8692	-0.5417	-0.7718	1.2161
1	SB	9	-1.2389	-0.0008	1.0981	-0.4586	-1.2337	-0.0996
1	SB	10	0.5575	1.2573	2.6397	-0.4586	-1.2337	-0.0996
1	SB	11	-0.8797	-0.3908	-0.2479	-1.0405	-1.2337	-0.0996
2	BB	12	-0.1611	2.3950	-0.7656	-0.2924	0.3044	1.7510
2	BB	13	-0.3409	-0.6526	-0.6046	-0.1261	0.3044	1.7510
2	BB	14	-0.1609	-0.7754	-0.7426	-0.7911	0.3044	1.7510
2	BB	15	-0.5204	-0.0700	0.3273	1.2038	0.3044	1.7510
2	BB	16	-1.7778	-1.5100	0.0512	3.0325	0.3044	1.7510
2	DZ	17	0.5575	0.9021	-0.5701	0.1233	0.0708	-0.8201
2	DZ	18	-0.1611	-0.4388	0.2237	-0.7080	0.0708	-0.8201
2	DZ	19	-0.3407	-1.3978	-0.2249	-0.7911	0.0708	-0.8201
2	DZ	20	0.5575	-2.0149	-0.6506	-1.3729	0.0708	-0.8201
2	DZ	21	-0.8797	0.2049	-0.6276	-1.2898	0.0708	-0.8201
2	MZ	22	1.0964	0.5799	-1.1683	-0.6248	2.4772	-0.8201
2	MZ	23	1.4559	0.5495	-0.8117	-0.5417	2.4772	-0.8201
2	MZ	24	1.8148	1.6065	2.2715	1.9519	2.4772	-0.8201
2	WH	25	1.0964	0.6881	1.4777	0.8713	0.1873	-0.8283
2	WH	26	1.2760	-0.1902	0.6149	-0.2924	0.1873	-0.8283
2	WH	27	1.0964	0.1648	0.2237	-0.2924	0.1873	-0.8283
2	WH	28	0.3780	-1.7400	-0.1214	0.3726	0.1873	-0.8283
2	WH	29	1.2760	0.4613	2.1220	0.2895	0.1873	-0.8283

Table S6. Peatland scale habitat metrics measured for each peatland (Ptld), in each system (Sys). Values were standardized by subtracting the mean and dividing by the standard deviation. Codes correspond to those in Table S3: S_{cl} = patch size of the cluster, D_{cl} = inter-patch distance among clusters, A_{ptld} = amount of habitat in the peatland, and I_{ptld} = isolation of the peatland.

Sys	Ptld	S_{cl}	D_{cl}	A_{ptld}	I_{ptld}
1	MN	-0.6673	-0.0492	-0.7647	-0.2833
1	RSB	-1.1747	-1.0502	-0.7008	1.1373
1	SB	1.4665	-0.7721	-1.1056	-0.1118
2	BB	-0.5637	0.5427	0.2433	1.6467
2	DZ	-0.5972	1.7917	0.0382	-0.7938
2	MZ	0.4774	-0.8175	2.1497	-0.7938
2	WH	1.0591	0.3545	0.1400	-0.8012

Table S7. Global models used to generate candidate model sets in the plant, cluster, and peatland scale datasets.

Scale	Global model
Plant	$F_{ST} = S_{lf} + D_{lf} + A_{pl} + I_{pl} + A_{cl} + I_{cl} + A_{ptld} + I_{ptld} + D_{lf}:A_{pl}$ + (1|cluster) + (1|peatland) + (1|system)
Cluster	$F_{ST} = S_{pl} + D_{pl} + A_{cl} + I_{cl} + A_{ptld} + I_{ptld} + D_{pl}:A_{cl}$ + (1|peatland) + (1|system)
Peatland	$F_{ST} = S_{cl} + D_{cl} + I_{ptld} + A_{ptld} + D_{cl}:A_{ptld}$ + (1|system)

Table S8. Summary of plant scale genetic data with the average number of alleles (N_A), observed (H_O) and expected (H_E) levels of heterozygosity, inbreeding coefficient (F_{IS}), and F_{ST} values (bolded values P < 0.05).

Peatland (n = 7)	Cluster (n = 27)	Plant (n = 65)	Number of individuals (n = 921)	N_A	H_O	H_E	F_{IS}	F_{ST}	P
MN	1	1	11	2.533	0.558	0.453	-0.218	**0.0970**	0.0180
MN	1	2	14	3.367	0.625	0.534	-0.170	-0.0080	0.5520
MN	1	3	13	2.900	0.589	0.470	-0.277	0.0250	0.2370
MN	2	4	12	2.767	0.592	0.453	-0.295	**0.0990**	0.0140
MN	3	5	15	3.167	0.547	0.499	-0.106	**0.0510**	0.0470
MN	3	6	15	3.067	0.613	0.509	-0.195	-0.0060	0.5330
MN	3	7	15	3.133	0.620	0.498	-0.241	0.0430	0.0830
MN	4	8	11	2.767	0.600	0.453	-0.339	0.0370	0.1850
MN	4	9	12	2.967	0.580	0.483	-0.226	-0.0180	0.6360
MN	4	10	15	3.400	0.580	0.494	-0.159	0.0073	0.3491
MN	5	11	13	2.833	0.636	0.481	-0.309	0.0445	0.1229
MN	5	12	12	2.900	0.575	0.470	-0.233	0.0559	0.0851
RSB	6	13	15	3.033	0.575	0.465	-0.236	0.0329	0.1094
RSB	6	14	15	3.467	0.573	0.491	-0.159	-0.0433	0.9809
RSB	7	15	15	3.367	0.647	0.494	-0.314	0.0195	0.2247
RSB	7	16	15	3.467	0.607	0.512	-0.204	0.0251	0.1600
RSB	7	17	15	3.467	0.580	0.517	-0.115	0.0326	0.0952
RSB	8	18	15	2.633	0.613	0.469	-0.307	**0.1307**	0.0010
RSB	8	19	15	3.067	0.607	0.469	-0.298	**0.0866**	0.0079
RSB	8	20	15	2.967	0.493	0.459	-0.072	0.0132	0.2741
SB	9	21	15	3.233	0.587	0.487	-0.173	0.0252	0.1666
SB	9	22	15	3.600	0.607	0.536	-0.124	-0.0303	0.9196
SB	10	23	15	3.200	0.553	0.464	-0.189	0.0404	0.0780
SB	10	24	15	3.467	0.513	0.508	-0.003	-0.0097	0.6300
SB	10	25	15	3.600	0.653	0.572	-0.145	-0.0326	0.9245
SB	11	26	13	2.967	0.573	0.480	-0.187	-0.0159	0.6470
SB	11	27	15	3.300	0.567	0.503	-0.133	**0.0491**	0.0351
BB	12	28	11	2.933	0.547	0.468	-0.193	**0.0797**	0.0183
BB	12	29	15	3.400	0.627	0.533	-0.178	0.0184	0.2247
BB	13	30	15	2.533	0.447	0.345	-0.321	**0.1107**	0.0015
BB	13	31	15	3.133	0.640	0.508	-0.260	**0.0877**	0.0075
BB	13	32	15	3.033	0.627	0.472	-0.337	**0.0971**	0.0093
BB	14	33	13	3.000	0.577	0.485	-0.166	0.0328	0.1649
BB	14	34	14	3.000	0.555	0.498	-0.124	0.0495	0.0762
BB	15	35	12	3.167	0.598	0.506	-0.184	0.0167	0.2755

BB	15	36	13	3.000	0.576	0.495	-0.182	0.0298	0.1710
BB	15	37	13	2.733	0.591	0.453	-0.306	0.0166	0.2937
DZ	16	38	15	3.500	0.567	0.519	-0.096	-0.0315	0.9255
DZ	16	39	15	3.000	0.527	0.453	-0.185	0.0099	0.3341
DZ	16	40	15	3.233	0.540	0.486	-0.095	0.0409	0.0864
DZ	17	41	15	3.367	0.600	0.486	-0.230	0.0151	0.2526
DZ	17	42	15	3.200	0.513	0.471	-0.101	-0.0103	0.6094
DZ	18	43	15	3.067	0.580	0.470	-0.221	-0.0046	0.5030
DZ	18	44	15	3.133	0.533	0.455	-0.184	**0.0806**	0.0085
DZ	18	45	15	3.100	0.580	0.488	-0.192	-0.0072	0.5326
DZ	19	46	13	3.267	0.593	0.516	-0.144	-0.0037	0.5038
DZ	19	47	15	3.367	0.560	0.499	-0.118	0.0084	0.3392
DZ	19	48	15	2.933	0.620	0.451	-0.395	**0.0769**	0.0291
MZ	20	49	15	3.100	0.573	0.495	-0.154	0.0188	0.2349
MZ	20	50	15	3.100	0.567	0.473	-0.191	0.0299	0.1329
MZ	20	51	12	3.000	0.571	0.480	-0.101	0.0025	0.4187
MZ	21	52	15	3.033	0.513	0.473	-0.066	-0.0323	0.8950
WH	22	53	13	3.000	0.582	0.487	-0.191	0.0101	0.3450
WH	22	54	13	2.800	0.552	0.446	-0.241	0.0249	0.2166
WH	23	55	15	3.067	0.507	0.498	-0.023	0.0097	0.3279
WH	23	56	15	3.067	0.633	0.480	-0.333	0.0408	0.1012
WH	23	57	15	3.200	0.607	0.509	-0.183	0.0036	0.3970
WH	24	58	15	3.467	0.647	0.521	-0.245	0.0081	0.3354
WH	24	59	12	3.000	0.660	0.492	-0.317	-0.0091	0.5197
WH	24	60	13	2.800	0.611	0.465	-0.329	**0.0767**	0.0392
WH	25	61	13	2.867	0.504	0.466	-0.090	-0.0340	0.8603
WH	25	62	15	3.100	0.556	0.493	-0.140	**0.0588**	0.0494
WH	26	63	15	3.067	0.520	0.473	-0.102	0.0229	0.1861
WH	26	64	15	2.833	0.507	0.439	-0.153	0.0496	0.0642
WH	26	65	15	3.300	0.580	0.536	-0.069	-0.0343	0.9018

Table S9. Summary of cluster scale genetic data with the average number of alleles (N_A), observed (H_O) and expected (H_E) levels of heterozygosity, inbreeding coefficient (F_{IS}), and F_{ST} values (bolded values $P < 0.05$).

Peatland (n = 7)	Cluster (n = 29)	Number of individuals (n = 1,165)	N_A	H_O	H_E	F_{IS}	F_{ST}	P
MN	1	38	3.933	0.595	0.545	-0.091	0.0160	0.0910
MN	2	32	4.267	0.588	0.546	-0.070	-0.0066	0.6768
MN	3	45	4.433	0.593	0.548	-0.080	0.0160	0.0548
MN	4	38	4.267	0.591	0.522	-0.147	0.0132	0.1092
MN	5	37	4.100	0.601	0.537	-0.108	**0.0287**	0.0202
RSB	6	42	4.500	0.569	0.509	-0.112	**0.0179**	0.0277
RSB	7	45	4.867	0.611	0.551	-0.111	0.0055	0.2172
RSB	8	45	4.400	0.571	0.525	-0.083	**0.0188**	0.0281
SB	9	42	4.533	0.606	0.555	-0.084	-0.0089	0.8636
SB	10	45	4.833	0.573	0.552	-0.033	0.0090	0.1196
SB	11	38	4.633	0.595	0.542	-0.090	-0.0010	0.4800
BB	12	35	4.600	0.594	0.548	-0.083	0.0174	0.0682
BB	13	35	4.600	0.590	0.551	-0.073	**0.0242**	0.0223
BB	14	45	4.467	0.571	0.502	-0.148	**0.0871**	0.0001
BB	15	37	4.133	0.545	0.520	-0.048	**0.0399**	0.0017
BB	16	38	4.467	0.582	0.534	-0.099	**0.0241**	0.0270
DZ	17	36	4.433	0.478	0.506	0.072	0.0069	0.2276
DZ	18	45	4.733	0.544	0.524	-0.040	**0.0200**	0.0268
DZ	19	42	4.567	0.557	0.505	-0.102	-0.0003	0.4336
DZ	20	45	4.733	0.564	0.511	-0.109	0.0039	0.2842
DZ	21	43	4.700	0.596	0.534	-0.122	-0.0012	0.4911
MZ	22	42	4.700	0.566	0.527	-0.002	0.0023	0.3520
MZ	23	38	4.100	0.560	0.505	-0.105	**0.0384**	0.0054
MZ	24	30	3.700	0.617	0.522	-0.186	**0.0396**	0.0175
WH	25	36	3.800	0.576	0.504	-0.135	-0.0029	0.5499
WH	26	45	4.400	0.582	0.536	-0.083	-0.0032	0.5970
WH	27	40	4.533	0.638	0.534	-0.184	**0.0368**	0.0057
WH	28	41	4.433	0.508	0.521	0.014	-0.0045	0.6480
WH	29	45	4.400	0.536	0.522	-0.028	0.0030	0.3176

Table S10. Summary of peatland scale genetic data with the average number of alleles (N_A), observed (H_O) and expected (H_E) levels of heterozygosity, inbreeding coefficient (F_{IS}), and F_{ST} values (bolded values $P < 0.05$).

Peatland (n = 7)	Number of individuals (n = 1,199)	N_A	H_O	H_E	F_{IS}	F_{ST}	P
MN	190	5.620	0.593	0.559	-0.059	**0.0089**	0.0026
RSB	132	6.133	0.584	0.545	-0.066	**0.0058**	0.0268
SB	125	5.800	0.589	0.563	-0.040	0.0037	0.1043
BB	190	6.560	0.578	0.562	-0.032	**0.0107**	0.0004
DZ	211	6.540	0.548	0.531	-0.029	**0.0074**	0.0053
MZ	144	6.125	0.571	0.540	0.013	**0.0169**	0.0005
WH	207	5.760	0.568	0.539	-0.051	**0.0107**	0.0004

Table S11. Pearson correlation coefficients indicating the correlation between predictor variables used in plant scale models (significance levels $^{*}P < 0.05$, $^{**} < 0.01$, $^{***} < 0.001$).

Variable	D_{lf}	I_{pl}	I_{cl}	I_{ptld}	A_{pl}	A_{cl}	A_{ptld}
S_{lf}	0.425^{***}	0.030	-0.044	-0.154	0.030	0.157	0.302^{**}
D_{lf}		0.138	-0.030	-0.102	-0.009	0.191	0.285^{**}
I_{pl}			-0.091	-0.125	-0.004	0.078	-0.067
I_{cl}				0.276^{**}	-0.060	0.343^{***}	0.090
I_{ptld}					-0.315^{**}	-0.294^{**}	-0.334^{***}
A_{pl}						0.224^{*}	0.296^{**}

Table S12. Pearson correlation coefficients indicating the correlation between predictor variables used in cluster scale models (significance levels $^{*}P < 0.05$, $^{**} < 0.01$, $^{***} < 0.001$).

Variable	D_{pl}	I_{cl}	I_{ptld}	A_{cl}	A_{ptld}
S_{pl}	0.227	-0.150	-0.508^{**}	0.365	0.572^{**}
D_{pl}		0.053	-0.076	0.288	0.166
I_{cl}			0.334	0.290	0.147
I_{ptld}				-0.276	-0.198
A_{cl}					0.007

Table S13. Pearson correlation coefficients indicating the correlation between predictor variables used in peatland scale models (significance levels $^{*}P < 0.05$, $^{**} < 0.01$, $^{***} < 0.001$).

Variable	D_{cl}	I_{ptld}	A_{ptld}
S_{cl}	-0.209	-0.499	0.014
D_{cl}		-0.209	0.194
I_{ptld}			-0.344

Table S14. Summary of model selection statistics for candidate models at the plant, cluster, and peatland scales, with log likelihood (logLik) statistics, corrected Akaike information criterion (AIC_c), Δ_i AIC_c, and Akaike weights (w_i). Models are ranked according to AIC_c and may be compared by $\Delta_i AIC_c$. Models within $\Delta_i AIC_c < 4$ are presented.

Model	logLik	AIC_c	$\Delta_i AIC_c$	w_i
Plant scale				
$I_{cl} + I_{ptld}$	111.132	-232.00	0.00	0.063
$D_{lf} + I_{cl} + I_{ptld}$	107.362	-231.80	0.20	0.057
$I_{pl} + I_{cl} + I_{ptld}$	107.770	-231.67	0.33	0.053
I_{ptld}	113.440	-231.27	0.73	0.044
$D_{lf} + I_{pl} + I_{cl} + I_{ptld}$	103.906	-231.26	0.74	0.043
$I_{pl} + I_{ptld}$	109.751	-230.34	1.66	0.027
$A_{cl} + I_{ptld}$	109.718	-230.22	1.78	0.026
$S_{lf} + I_{cl} + I_{ptld}$	106.890	-230.15	1.85	0.025
$A_{pl} + I_{cl} + I_{ptld}$	103.514	-229.72	2.28	0.020
$S_{lf} + I_{pl} + I_{cl} + I_{ptld}$	109.404	-229.62	2.38	0.019
$D_{lf} + I_{ptld}$	106.926	-229.60	2.40	0.019
$A_{cl} + I_{cl} + I_{ptld}$	106.716	-229.41	2.59	0.017
$A_{pl} + I_{pl} + I_{cl} + I_{ptld}$	109.309	-229.40	2.60	0.017
$D_{lf} + A_{pl} + I_{cl} + I_{ptld}$	103.070	-229.30	2.70	0.016
$S_{lf} + I_{ptld}$	106.019	-229.21	2.79	0.016
$D_{lf} + A_{cl} + I_{cl} + I_{ptld}$	102.977	-229.12	2.88	0.015
$I_{pl} + A_{cl} + I_{ptld}$	103.135	-229.10	2.90	0.015
$S_{lf} + D_{lf} + I_{cl} + I_{ptld}$	103.538	-229.08	2.92	0.015
$I_{pl} + A_{cl} + I_{cl} + I_{ptld}$	106.860	-228.94	3.06	0.014
$I_{cl} + A_{ptld} + I_{ptld}$	103.350	-228.93	3.07	0.014
$D_{lf} + A_{pl} + I_{pl} + I_{cl} + I_{ptld}$	109.100	-228.90	3.10	0.013
$A_{pl} + I_{ptld}$	102.991	-228.77	3.23	0.013
$D_{lf} + I_{cl} + A_{ptld} + I_{ptld}$	109.004	-228.75	3.25	0.012
$A_{ptld} + I_{ptld}$	99.544	-228.57	3.43	0.011
$D_{lf} + I_{pl} + I_{ptld}$	105.673	-228.53	3.47	0.011
$I_{pl} + I_{cl} + A_{ptld} + I_{ptld}$	103.502	-228.52	3.48	0.011
$S_{lf} + D_{lf} + I_{pl} + I_{cl} + I_{ptld}$	99.676	-228.47	3.53	0.011
$S_{lf} + I_{pl} + I_{ptld}$	105.638	-228.43	3.57	0.011
$D_{lf} + I_{pl} + A_{cl} + I_{cl} + I_{ptld}$	99.593	-228.38	3.62	0.010
$D_{lf} + A_{cl} + I_{ptld}$	105.585	-228.28	3.72	0.010
$S_{lf} + A_{cl} + I_{ptld}$	105.506	-228.09	3.91	0.009
Cluster scale				
$A_{ptld} + I_{ptld}$	64.569	-142.60	0.00	0.227
$S_{pl} + I_{ptld}$	64.582	-142.00	0.60	0.169

$S_{pl} + A_{ptld} + I_{ptld}$	60.905	-141.06	1.54	0.105
$I_{cl} + A_{ptld} + I_{ptld}$	60.150	-139.89	2.71	0.059
$S_{pl} + A_{cl} + I_{ptld}$	60.038	-139.35	3.25	0.045
$A_{cl} + A_{ptld} + I_{ptld}$	59.767	-139.10	3.50	0.040
$D_{pl} + A_{ptld} + I_{ptld}$	59.734	-139.09	3.51	0.039
I_{ptld}	66.680	-138.70	3.90	0.032
Peatland scale				
$S_{cl} + D_{cl} + A_{ptld} + D_{cl}:A_{ptld}$	7.541	-182.0	0.00	0.994
